# A Novel Calix[4]Crown-Based 1,3,4-Oxadiazole as a Fluorescent Chemosensor for Copper(II) Ion Detection

**DOI:** 10.3389/fchem.2021.766442

**Published:** 2021-11-12

**Authors:** Chun Sun, Siyi Du, Tianze Zhang, Jie Han

**Affiliations:** Key Laboratory of Advanced Energy Materials Chemistry (Ministry of Energy), College of Chemistry, Nankai University, Tianjin, China

**Keywords:** calix[4]crown, 1,3-alternate conformation, 1,3,4-oxadiazole, copper (II) detection, fluorescent chemosensor

## Abstract

The synthesis and characterization of a novel florescent chemosensor **1** with two different types of cationic binding sites have been reported in this work, which is a calix[4]crown derivative in 1,3-alternate conformation bearing two 2-phenyl-5-(4-dimethylaminopyenyl)-1,3,4-oxadiazole units. The recognition behaviors of **1** in dichloromethane/acetonitrile solution to alkali metal ions (Na^+^ and K^+^), alkaline earth metal ions (Mg^2+^ and Ca^2+^), and transition metal ions (Co^2+^, Ni^2+^, Zn^2+^, Cd^2+^, Cu^2+^, Mn^2+^, and Ag^+^) have been investigated by UV-Vis and fluorescence spectra. The fluorescence of **1** might be quenched selectively by Cu^2+^ due to the photo-induced electron transfer mechanism, and the quenched emission from **1** could be partly revived by the addition of Ca^2+^ or Mg^2+^; thus, the receptor **1** might be worked as an on–off switchable fluorescent chemosensor triggered by metal ion exchange.

## Introduction

As the third most abundant transition metal ion after zinc and iron in the human body, copper is required by many living organisms for normal physiological processes (Turski and Thiele, 2009; Cotruvo Jr et al., 2015). Maintaining optimal concentration of Cu^2+^ ion for living cells is an essential factor to keep the normal functioning of enzymes and intracellular metabolic balance. Thus, the development of new fluorescent chemosensors for Cu^2+^ ion has drawn continuous interest during the past decades. The main progress in this area has been well reviewed (Cao et al., 2019; Sivaraman et al., 2018; Udhayakumari et al., 2017; Liu et al., 2017), and many fluorescent chemosensors for Cu^2+^ ion based on various fluorophores such as coumarin (Zhang et al., 2019), Bodipy (Ömeroğlu et al., 2021), rhodamine (Fernandes and Raimundo, 2021), Schiff base (Singh et al., 2020), pyrene (Kowser et al., 2021), and 1,3,4-oxadiazole (Wang, et al., 2018) have been reported by different research groups. Among these fluorescent chemosensors, the 1,3,4-oxadiazoles have drawn special interest due to their electron-deficient nature, high photoluminescence quantum yield, and excellent chemical stability, and have found practical applications in the fields of organic light-emitting diodes (Meng et al., 2020) and liquid crystals (Han et al., 2013; Han et al., 2015; Han et al., 2018). In addition, the nitrogen and oxygen atoms of the 1,3,4-oxadiazole unit can provide potential coordination sites with metal ions, which makes it usable as a signaling component in fluorescent chemosensors.

**SCHEME 1:**
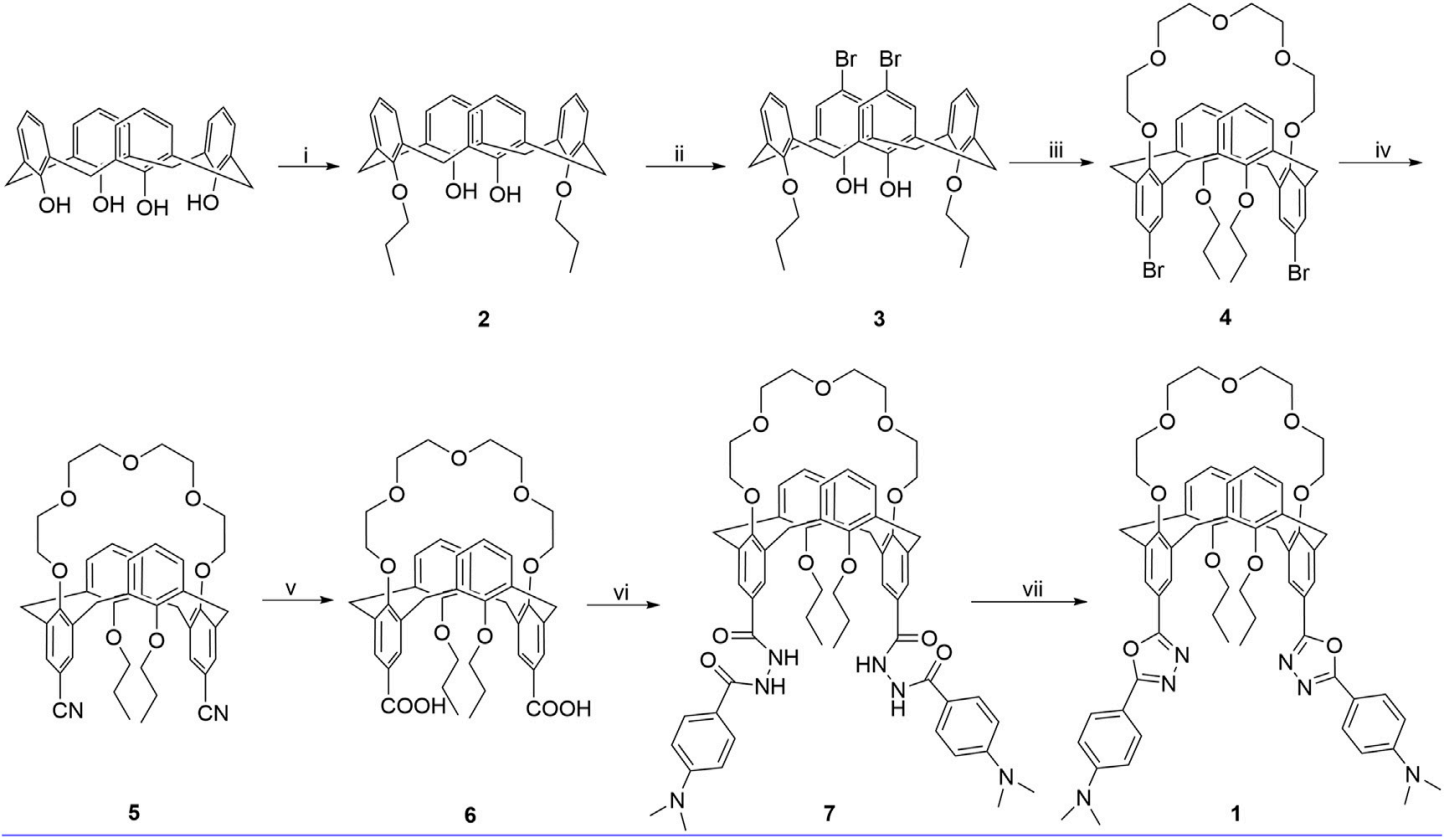
Synthetic route for **1**, reagents, and conditions: (i) 1-iodopropane, K_2_CO_3_, CH_3_CN, reflux, 24 h; (ii) Br_2_, 0°C, 3 h; (iii) tetraethylene glycol ditosylate, Cs_2_CO_3_, CH_3_CN, reflux, 72 h; (iv) (1) CuCN, NMP, 180°C, 5 h; (2) FeCl_3_, 2 M HCl, 100°C, 1 h; (v) KOH, ethanol, reflux, 24 h; (vi) (1) SOCl_2_, toluene, reflux, 5 h; (2) 4-(dimethylamino)benzohydrazide, pyridine, r. t., 12 h; (vii) POCl_3_, reflux, 12 h.

Calixarenes, as one kind of the most important super-molecules, have been widely used in design of fluorescent chemosensors for ions and neutral molecules due to their outstanding features such as preorganized binding sites, easy derivatization, and flexible three-dimensional structures (Kim et al., 2012; An et al., 2019; Miranda et al., 2019; Noruzi et al., 2019; Chen et al., 2020). Many calixarene-based fluorescent chemosensors for transition metal ions have been reported in recent years (Ma et al., 2015). However, the fluorescent switchable chemosensors triggered by different ions are quite few (Chung et al., 2007), which remains a challenge in the field of supramolecular chemistry. Herein, as part of our continuous research in the design and synthesis of new fluorescent chemosensors (Liu et al., 2021; Xie et al., 2016; Han et al., 2012), we utilize the 1,3-alternate calix[4]crown scaffold to construct an on–off switchable fluorescent chemosensor **1** in this work. The synthetic route for **1** is shown in Scheme 1. There are quite a number of chemosensors based on various macrocycles for copper detection reported in literatures (Lvova, et al., 2018; Doumani, et al., Kamei, et al., 2021), in which the macrocyles often only worked as receptors for Cu^2+^ ions. In contrast, the chemosensor **1** in this work is special in that it has two kinds of macrocycles: one is from the 1,3-alternate calixarene, which provides a three-dimensional scaffold with two appending 1,3,4-oxadiazole units as both signaling component and fluorophore; the other is from the calix[4]crown, which can bind the Mg^2+^ or Ca^2+^ ions and has an allosteric effect on the 1,3,4-oxadiazole units on opposite rings. The selective binding of 1,3,4-oxadiazole with Cu^2+^ ions results in the fluorescence quenching, while the binding of calix[4]crown with Mg^2+^ or Ca^2+^ ions can partly revive the fluorescence consequently. Thus, the compound **1** might work as a new type of switchable off–on fluorescent chemosensor.

## Materials and Methods

25,27-Dihydroxy-26,28-dipropoxycalix[4]arene **2** and 5,17-dibromo-25,27-dihydroxy-26,28-dipropoxycalix[4]arene **3** were synthesized according to the literature procedures (Hobzova, 2010). Dichloromethane and acetonitrile used for photophysical studies were of spectrometric grade. All the other chemicals and solvents were of analytical grade and used as received from commercial sources. The solutions of metal ions were all prepared from their perchlorate salts. Column chromatography was performed on silica gel (200–300 mesh).

Solution ^1^H NMR (Proton Nuclear Magnetic Resonance) and ^13^C NMR (Carbon-13 Nuclear Magnetic Resonance) spectra were recorded on a Bruker AV400 spectrometer and the chemical shifts are quoted in parts per million (ppm) relative to tetramethylsilane (TMS) as an internal standard. ESI-HRMS (Electrospray Ionization-High Resolution Mass Spectrometry) data were obtained with a FTICR-MS mass spectrometer. Melting points were determined with an X-4 melting point apparatus, and the thermometer was uncorrected. Data for single x-ray structure were collected on a SMART1000 CCD-X diffractometer with graphite-monochromatized MoKα x-ray radiation (*λ* = 0.71073 Å) and Saturn CCD area detector. The x-ray crystal structure of 4 was solved by direct method and expanded using Fourier synthesis technique. No absorption correction was done. The non-hydrogen atoms were refined anisotropically. Hydrogen atoms were refined using riding model. Structural refinement based on full-matrix least-squares refinement on |F|^2^ was performed by using Crystal Structure or SHELXL97 suite program (Sheldrick, 1997).

### Synthesis of 4

A mixture of **3** (9.23 g, 13.9 mmol) and Cs_2_CO_3_ (11.30 g, 34.7 mmol) in MeCN (700 ml) under nitrogen was stirred at reflux for 30 min and then a solution of the tetraethylene glycol ditosylate (7.85 g, 15.6 mmol) in MeCN (40 ml) was added during an hour. The mixture was refluxed for 72 h and allowed to cool to room temperature. After evaporation of the solvent *in vacuo*, the residue was taken up in CH_2_Cl_2_ (30 ml × 3) and the resultant solution was washed with 1 mol/L HCl (30 ml) and brine (30 ml × 2). The organic layer was dried over MgSO_4_ and evaporated *in vacuo*. Recrystallization of the residue from CH_2_Cl_2_/MeOH gave **4** a pale-yellow solid. Yield, 75%. Mp: 230–232 °C. ^1^H NMR (400 MHz, CDCl_3_) δ 7.17 (s, 4H), 7.08 (d, J = 8.0 Hz, 4H), 6.87 (t, J = 8.0 Hz, 2H), 3.78 (d, J = 4.0 Hz, 8H), 3.54 (s,8H), 3.45 (t, J = 8.0 Hz, 4H), 3.25–3.20 (m, 4H), 3.15 (m, 4H), 1.31 (m, 4H), 0.78 (t, J = 7.5 Hz, 6H). ^13^C NMR (101 MHz, CDCl_3_) δ 156.97, 155.14, 136.29, 133.81, 132.17, 129.70, 122.77, 115.15, 72.59, 72.09, 70.50, 70.13, 69.03, 37.89, 22.53, 10.23. HRMS-MALDI calculated for C_42_H_48_Br_2_O_7_ [M + Na] ^+^ 847.1639, found 847.1652.

### Synthesis of 5

Under nitrogen, a mixture of **4** (10.17 g, 12.3 mmol) and cuprous cyanide (7.68 g, 86.4 mmol) in 20 ml of 1-methyl-2-pyrrolidinone was stirred at 180°C for 4 h. Then, the reaction mixture was cooled slowly to 100°C, and a solution of 23.23 g (86.4 mmol) of FeCl_3_·6H_2_O in 5 ml of concentrated hydrochloride and 25 ml of water was added to the reaction mixture. The reaction mixture was further stirred at 100°C for 1 h and cooled to room temperature. The solid was filtered off and recrystallized from chloroform/hexane yielding 5.5 g of compound **5** as yellow solid. Yield 62%. ^1^H NMR (400 MHz, CDCl_3_) δ 7.36 (s, 4H), 7.10 (d, J = 7.4 Hz, 4H), 6.90 (s, 2H), 3.82 (d, J = 5.5 Hz, 8H), 3.55 (s, 8H), 3.46 (t, J = 7.4 Hz, 4H), 3.30 (t, J = 6.1 Hz, 4H), 3.17 (t, J = 6.1 Hz, 4H), 1.24 (m, 4H), 0.74 (t, J = 7.5 Hz, 6H).^13^C NMR (101 MHz, CDCl_3_) δ 159.98, 156.80, 135.72, 133.54, 133.05, 129.94, 123.05, 119.17, 106.08, 77.36, 72.52, 72.17, 70.56, 69.85, 69.13, 37.75, 22.81, 10.05; HRMS: calcd for C_44_H_48_N_2_O_7_ [M+NH_4_]^+^ 734.3800, found 734.3796.

### Synthesis of 6

A solution of 5.18 g (9.2 mmol) of KOH in 100 ml of water was added to the suspension of 1.32 g (1.80 mmol) of **5** in 20 ml of ethanol. The reaction mixture was heated under reflux for 24 h. After cooling, the aqueous solution hydrogen chloride (10% w/w) was added dropwise until the solution became slightly acidic. The precipitate was filtered off, washed with water, and dried to yield a yellow solid product **6** (1.33 g, 96%). Mp: 296–298°C. ^1^H NMR (400 MHz, CDCl_3_) δ 12.52 (s, 2H), 7.81–7.77 (m, 8H), 6.98–6.94 (m, 2H), 3.93–3.80 (m, 12H), 3.62 (s, 16H), 1.42–1.31 (m, 4H), 0.69 (t, J = 7.5 Hz, 6H).^13^C NMR (101 MHz, DMSO-*d*_*6*_) δ 167.03, 158.48, 156.72, 135.62, 133.71, 132.13, 130.41, 125.24, 122.05, 71.17, 70.07, 69.72, 40.15, 39.94, 39.73, 39.52, 39.31, 39.10, 38.89, 35.98, 21.74, 9.52. HRMS: calcd for C_44_H_50_O_11_ [M-H]^+^ 753.3280, found 753.3285.

### Synthesis of 1

To a round-bottomed flask was added **6** (80 mg, 0.1 mmol), 10 ml of toluene, and 1 ml of thionyl chloride, and the mixture was refluxed for 5 h. After cooling, the solvent and the excess of thionyl chloride were removed at reduced pressure to give the benzoyl chloride, which was added to a solution of 4-(dimethylamino)benzohydrazide (39 mg, 0.22 mmol) in 10 ml of dichloromethane and 0.1 ml of pyridine. The reaction mixture was stirred for 12 h at ambient temperature and filtered. The precipitate was washed with ethanol to give the bishydrazide **7** as white solid, which was used to the next step reaction without further purification. The intermediate compound **7** was added to POCl_3_ (5 ml), and the resultant solution was refluxed overnight under a nitrogen atmosphere. After the reaction mixture cooled to room temperature, it was poured into ice water and extracted with dichloromethane (3 × 10 ml). The combined organic layer was washed with water and brine, respectively. Then, the solvent was removed under reduced pressure, and the crude solid was purified by silica gel column chromatography using petroleum ether/ethyl acetate (1:1) as eluent affording the product **1** as white solids. Yield, 35%. Mp: 281–283°C. ^1^H NMR (400 MHz, CDCl_3_) δ 7.91 (d, J = 8.0 Hz, 4H), 7.81 (s, 4H), 7.15 (d, J = 8.0 Hz, 4H), 6.93 (s, 2H), 6.72 (d, J = 8.0 Hz, 4H), 3.93 (m, 8H), 3.59–3.53 (m, 12H), 3.31 (d, J = 5.2 Hz, 4H), 3.26 (d, J = 5.2 Hz, 4H), 3.05 (m, 12H), 1.25–1.19 (m, 4H), 0.60 (t, J = 7.4 Hz, 6H). ^13^C NMR (101 MHz, CDCl_3_) δ 164.80, 163.55, 158.76, 157.10, 152.20, 135.07, 133.83, 129.79, 128.32, 128.13, 122.75, 121.14, 118.35, 111.59, 111.27, 72.43, 72.24, 70.42, 70.14, 69.22, 40.11, 38.05, 22.43, 9.95. HRMS-ESI calculated for C_62_H_69_N_6_O_9_ [M + H] ^+^ 1,041.5120, found 1,041.5126. (Supplementary Figure S5, ESI).

### General Procedures for the UV/Vis and Fluorescence Experiments

UV-vis spectra were recorded on a Cary 3,010 spectrophotometer, and the resolution was set at 1 nm. Steady-state emission spectra were recorded on a Varian Cary Eclipse spectrometer. For all measurements of fluorescence spectra, excitation was set at 334 nm for complexation, and the excitation and emission slit width was set to be 2.5 nm. Fluorescence titration experiments were performed with CH_2_Cl_2_ solutions of compound **1** and varying concentrations of metal perchlorate in CH_3_CN solution. During all measurements, the temperature of the quartz sample cell and chamber was kept at 25°C.

## Results and Discussion

### Synthesis and Structural Analysis

**FIGURE 1:**
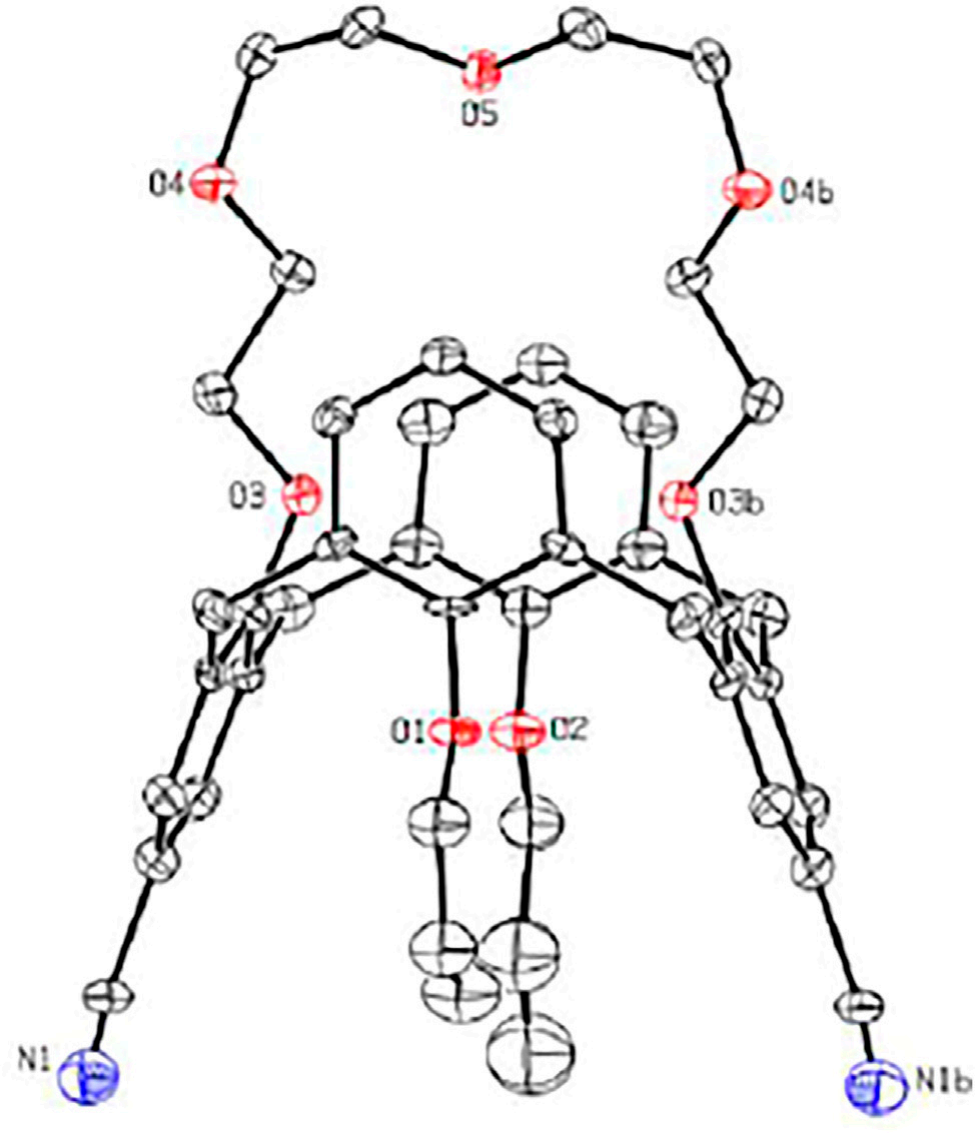
X-ray molecular structure of **5**.

As shown in Scheme 1, calix[4]arene **3** was reacted with tetraethylene glycol ditosylate in the presence of Cs_2_CO_3_ to successfully afford the calix[4]crown **3** in 75% yield. The substitution reaction of **4** with CuCN gave **5** in 62% yield, which was refluxed with KOH in ethanol and treated with hydrochloric acid solution, readily providing the carboxylic acid **6** in good yield. Then, the carboxylic acid **6** was reacted with thionyl chloride, and treated with benzyol hydrazine or 4-N,N′-dimethylaminobenzyol hydrazine to generate the intermediate bishydrazide **7**, which was used in the next step without purification and refluxed with phosphorus oxychloride to afford the target products **1**. Except for the calix[4]arene **3**, all of the intermediate calix[4]crowns **3**–**6** and the chemosensor **1** are in 1,3-alternate conformation, which were well established by ^1^H NMR and ^13^C NMR data (Supplementary Figures S1–S4, ESI). The 1,3-alternate conformation of **5** was further confirmed unambiguously by x-ray single crystal diffraction as shown in Figure 1. The x-ray crystallographic data are collected in Supplementary Table S1.

### UV-Vis Absorption and Fluorescence Spectra Analysis

**FIGURE 2:**
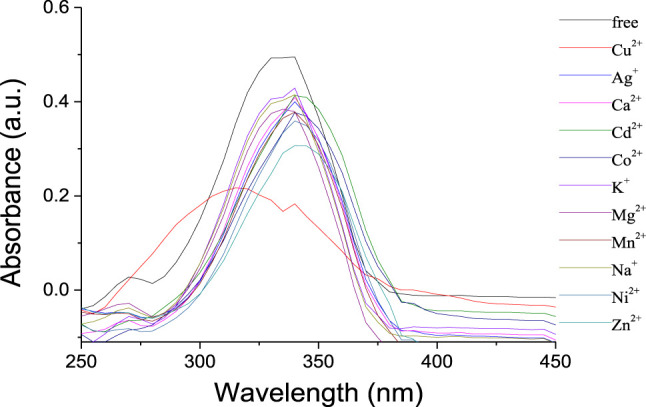
UV-vis spectra of **1** (1 × 10^−6^ mol/L) upon addition of metal ions (10 equiv) in CH_2_Cl_2_/CH_3_CN (1,000:1, v/v).

The selectivity of the receptor **1** toward different perchlorate salts, including Na^+^, K^+^, Mg^2+^, Ca^2+^, Co^2+^, Ni^2+^, Zn^2+^, Cd^2+^, Mn^2+^, Ag^+^, and Cu^2+^, was first investigated by UV-Vis spectroscopy. The UV-Vis absorption spectra for free **1** in CH_2_Cl_2_ solution showed an intense and structureless absorption band (*ε* = 4.94 × 10^5^ L/mol·cm) peaking at 340 nm (Figure 2), which might have resulted from the spin-allowed π-π* transitions involving the phenyloxadiazole moiety (Han et al., 2006). The addition of Cu^2+^ ions in the solution of **1** resulted in a significant decrease in the absorbance with an appreciable hypochromic shift of 20 nm. In contrast, only a slight decrease was observed upon addition of other metal ions mentioned above, which suggested that the selectivity of **1** toward Cu^2+^ is much higher than the other metal ions.

**FIGURE 3:**
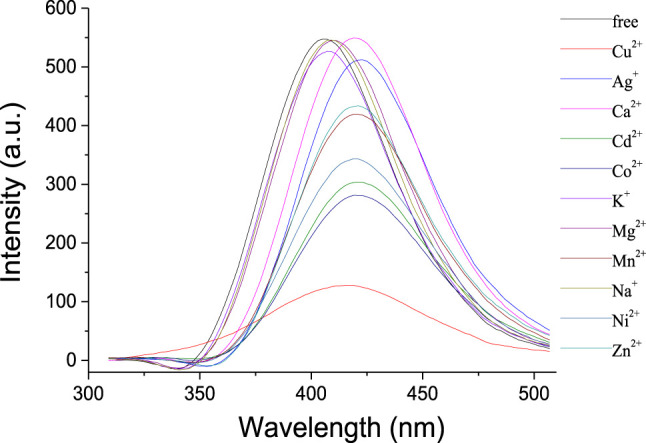
Fluorescence spectra (λ_exc_ = 334 nm, Slit = 2.5) of **1** (1 × 10^−6^ mol/L) upon addition of metal ions (10 equiv) in CH_2_Cl_2_/CH_3_CN (1,000:1, v/v).

Ion recognition ability of **1** was further studied by the fluorescence spectra. As shown in Figure 3, the receptor **1** exhibited a strong emission with λ_max_ at 405 nm in solution of CH_2_Cl_2_. Upon addition of Na^+^, K^+^, and Mg^2+^, respectively, almost no changes were observed in the intensity and shape of the emission spectra of **1**. It is noted that the addition of Ca^2+^ might slightly increase the intensity with a bathochromic shift of ca. 15 nm, perhaps because the complexation between the Ca^2+^ and the crown ether moiety changed the space distance of the two phenyloxadiazole units and the fluorescence changed consequently. Apparently, the fluorescence response of **1** toward transition metal ions was found to be more pronounced, and the addition of Co^2+^, Ni^2+^, Zn^2+^, and Ag^+^ could quench the emission of **1** in a different extent, accompanied by a concomitant red shift of ca. 14–17 nm. In contrast, the addition of Cu^2+^ significantly quenched the fluorescence of **1** under the same conditions as the aforementioned metal ions, suggesting that there is a strong interaction between 1,3,4-oxadiazole moieties of **1** and Cu^2+^ ion over the other metal ions.

**FIGURE 4:**
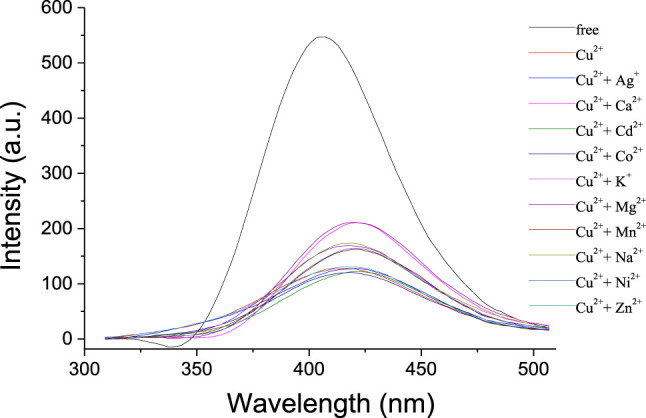
Fluorescence spectra (λ_exc_ = 334 nm, Slit = 2.5) of **1** (1 × 10^−6^ mol/L) and Cu^2+^ (10 equiv) upon addition of other metal ions (10 equiv) in CH_2_Cl_2_/CH_3_CN (1,000:1, v/v).

The fluorescence emission properties of **1** in the presence of Cu^2+^ and a competitive metal ion were measured to investigate the selective recognition for Cu^2+^. As shown in Figure 4, no apparent changes were observed in fluorescence intensity when 10 equivalent amounts of transition metal ions (Co^2+^, Ni^2+^, Zn^2+^, Cd^2+^, Mn^2+^, and Ag^+^) were added to the solution of **1** and Cu^2+^ (10 equiv). This suggested that the recognition for Cu^2+^ was not interrupted by the competitive transition metal ions; thus, the receptor **1** might act as a selective fluorescent chemosensor for Cu^2+^. The addition of alkali metal ions (Na^+^ and K^+^) to the solution of **1** and Cu^2+^ could increase the fluorescence intensity slightly, while the alkaline earth metal ions (Mg^2+^ and Ca^2+^) could revive the emission significantly.

**FIGURE 5:**
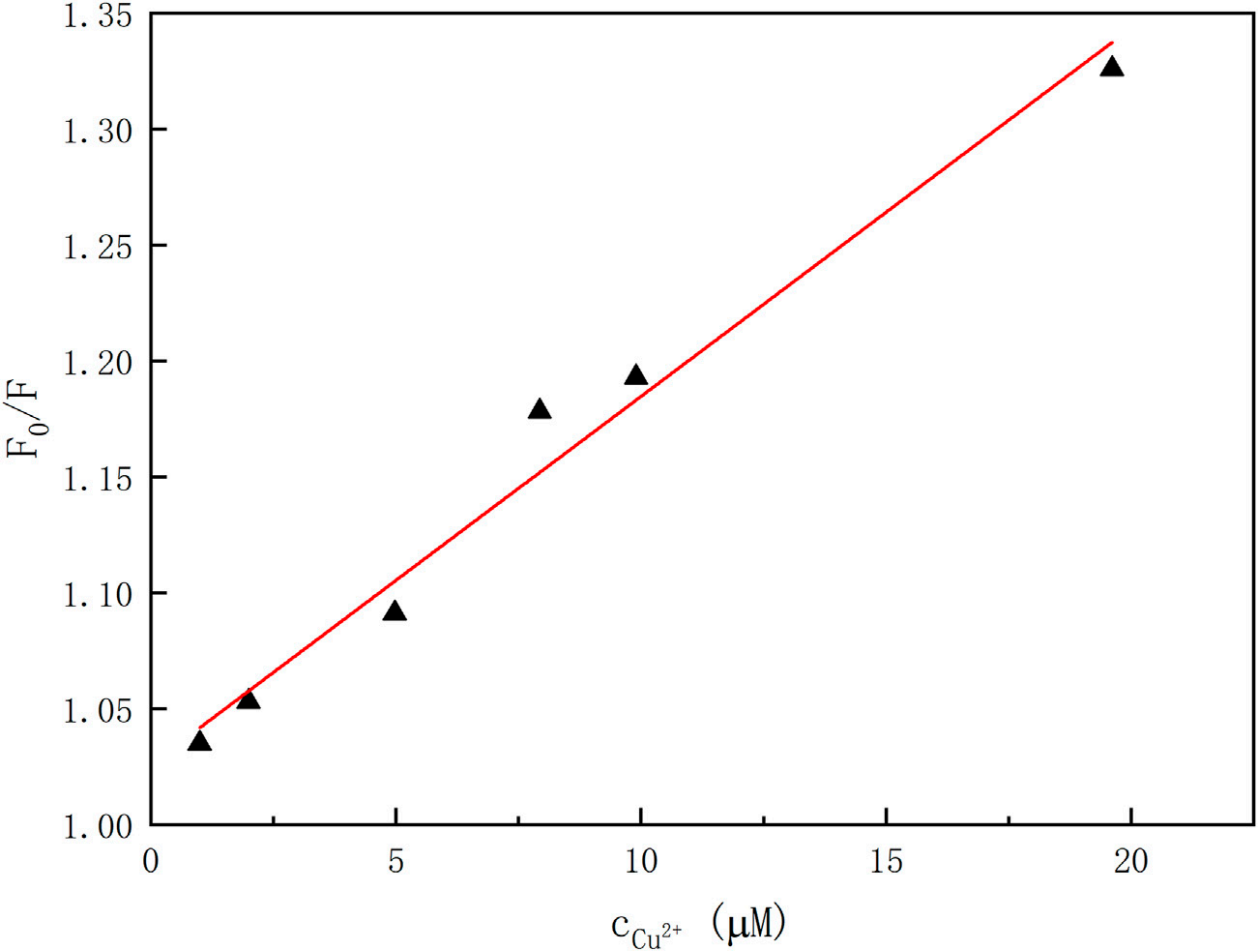
Plot of emission intensity versus the concentrations of Cu^2+^ ion (λ_em_ = 405 nm, λ_ex_ = 334 nm).

In order to elicit the binding property of the chemosensor **1** toward Cu^2+^ ion, fluorescence titration of **1** (1.0 × 10^–5^ mol/L) with Cu^2+^ ion (0–2 equiv) was carried out (Supplementary Figure S6). According to the fluorescence titration curves of **1** with Cu^2+^ ion at room temperatures, the association constant *K*_*a*_ was calculated as 1.6 × 10^–4^ L·mol^−1^ (*R* = 0.97526) for the **1**–Cu^2+^ complex by the Benesi–Hildebrand plot (Thordarson, 2011) (Figure 5). Moreover, the emission intensity of **1** is linearly proportional to the Cu^2+^ concentration in the range of 0–20 μM.

**FIGURE 6:**
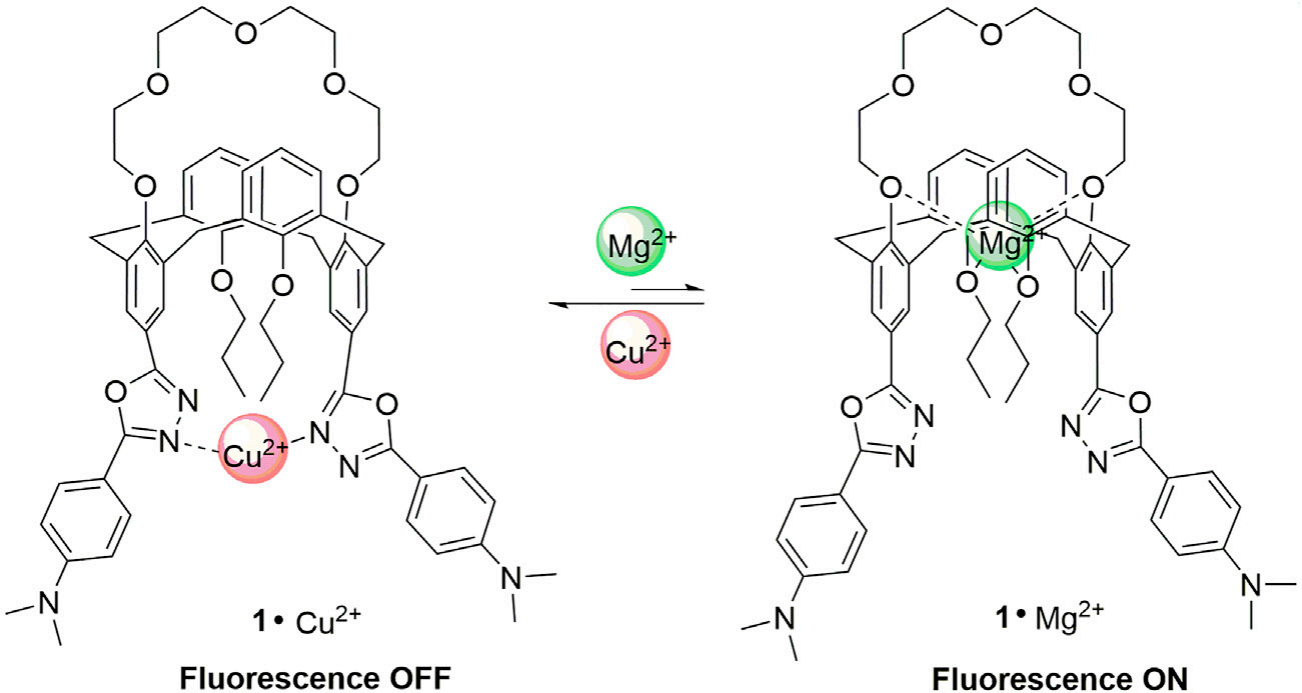
The complexation of **1** with Cu^2+^ and Mg^2+^ ions.

The fluorescence changes of **1** upon addition of Cu^2+^ and Mg^2+^ ions are displayed in Figure 6. The nitrogen atoms of the 1,3,4-oxadiazle units can bind with Cu^2+^ to form the complex **1**·Cu^2+^, and the paramagnetic nature of Cu^2+^ ion could strongly quench the fluorescence of the 1,3,4-oxadiazole units through the electron transfer mechanism, which is consistent to the results reported in literature (Han et al., 2012). In contrast, the polyether ring (crown-5 moiety) and the oxygens from the two propoxyl groups could provide coordination sites with the alkaline earth metal ions to form the complex **1**·Mg^2+^, which will change the molecular conformation as well as the space distance of the two 1,3,4-oxadiazole units. Consequently, the decomplexations between the 1,3,4-oxadiazoles and Cu^2+^ ions took place and resulted in the increase of the fluorescence. Thus, the receptor **1** might be acted as an on–off–on switchable fluorescent chemosensor triggered by the exchange of Cu^2+^ and Mg^2+^.

**FIGURE 7:**
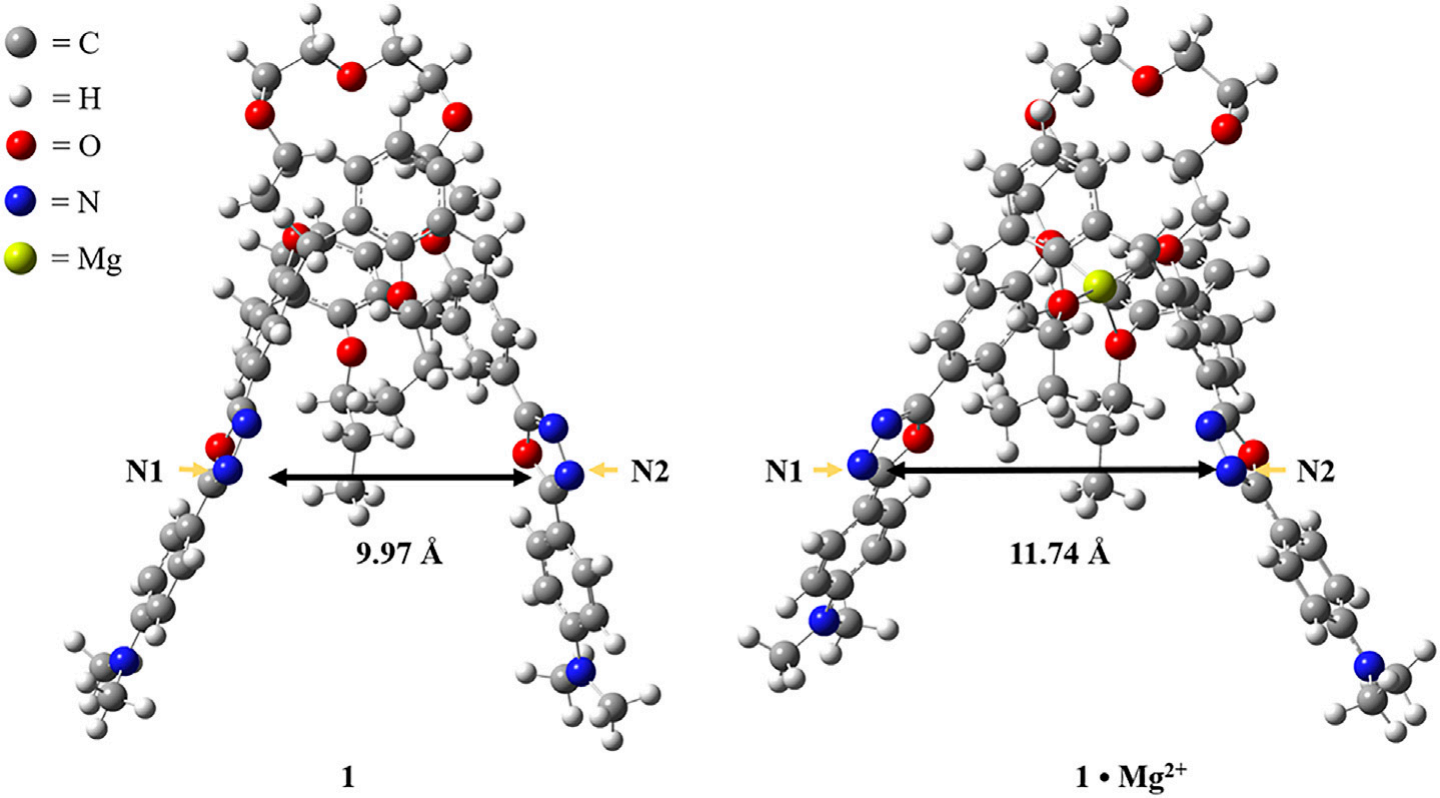
Computational optimized molecular structures of **1** and **1·Mg** ^**2+**^.

To gain a better understanding about the switchable fluorescence of the chemosensor **1**, DFT calculations with the GAUSSIAN 09 series of programs (Frisch et al., 2013) were carried out to analyze the molecular structures of **1** and **1·Mg** ^**2+**^, and DFT method B3-LYP with 6-31G(d) basis set was used for geometry optimizations (A. D. Becke, 1993). As shown in Figure 7, the distance between N1 and N2 in the free receptor **1** is 9.97 Ǻ, while the corresponding distance is 11.74 Ǻ in the complex **1·Mg** ^**2+**^, indicating that the molecular conformation changed simultaneously due to the allosteric effect (Kumar et al., 2012; Ni et al., 2013). The conformational change as well as the increase in distance makes it difficult for the chemosensor **1** to coordinate with Cu^2+^ ion to form the stable complex, which reasonably explains the fact that the addition of Mg^2+^ ions to the solution of **1** and Cu^2+^ can trigger the revival of fluorescence.

## Conclusion

In summary, we have designed a new type of fluorescent chemosensor based on a 1,3-alternate calix[4]crown with two different cationic binding sites. The 1,3,4-oxadiazole units could bind selectively with Cu^2+^ to form the complexation and resulted in the fluorescence quenching of the chemosensor. The presence of various transition metal ions does not interfere with the quenching process, while the alkaline earth metal ions Mg^2+^ might be entrapped by the crown-5 moiety and revive the fluorescence significantly due to the allosteric effect. As the chemosensor in this work is not soluble in water, it is difficult to investigate the Cu^2+^ ions’ detection under physiological conditions. Devising a water-soluble chemosensor for Cu^2+^ ions is in progress in our lab.

## Data Availability

The original contributions presented in the study are included in the article/Supplementary Material, further inquiries can be directed to the corresponding author.

## References

[B1] An L. Wang C. Han L. Liu J. Huang T. Zheng Y. (2019). Structural Design, Synthesis, and Preliminary Biological Evaluation of Novel Dihomooxacalix[4]arene-Based Anti-tumor Agent. Front. Chem. 7, 856. 10.3389/fchem.2019.00856 31921778 PMC6923765

[B2] Becke A. D. (1993). Density-functional Thermochemistry. III. The Role of Exact Exchange. J. Chem. Phys. 98, 5648. 10.1063/1.464913

[B3] Cao X. Li Y. Gao A. Yu Y. Zhou Q. Chang X. (2019). Multifunctional Fluorescent Naphthalimide Self-Assembly System for the Detection of Cu^2+^ and K^+^ and Continuous Sensing of Organic Amines and Gaseous Acids. J. Mater. Chem. C 7, 10589–10597. 10.1039/c9tc03243f

[B4] Chang K.-C. Su I.-H. Senthilvelan A. Chung W.-S. (2007). Triazole-modified Calix[4]crown as Novel Fluorescent On-Off Switchable Chemosensor. Org. Lett. 9, 3363–3366. 10.1021/ol071337+ 17650010

[B5] Chen Y.-J. Chen M.-Y. Lee K.-T. Shen L.-C. Hung H.-C. Niu H.-C. (2020). 1,3-Alternate Calix[4]arene Functionalized with Pyrazole and Triazole Ligands as a Highly Selective Fluorescent Sensor for Hg^2+^ and Ag+ Ions. Front. Chem. 6, 593261. 10.3389/fchem.2019.59326110.3389/fchem.2020.593261 PMC7688584 33282834

[B6] Cotruvo, Jr. J. A. Jr. Aron A. T. Ramos-Torres K. M. Chang C. J. (2015). Synthetic Fluorescent Probes for Studying Copper in Biological Systems. Chem. Soc. Rev. 44, 4400–4414. 10.1039/c4cs00346b 25692243 PMC4478099

[B7] Doumani N. Bou-Maroun E. Maalouly J. Tueni M. Dubois A. Bernhard C. (2019). A New pH-dependent Macrocyclic Rhodamine B-Based Fluorescent Probe for Copper Detection in white Wine. Sensors 19, 4514. 10.3390/s19204514 PMC6832540 31627384

[B8] Fernandes R. da. S. Raimundo I. M. Jr (2021). Development of a Reusable Fluorescent Nanosensor Based on Rhodamine B Immobilized in Stober Silica for Copper Ion Detection. Anal. Methods 13, 1970–1975. 10.1039/d1ay00168j 33913947

[B9] Frisch M. J. Trucks G. W. Schlegel H. B. Scuseria G. E. Robb M. A. Cheeseman J. R. (2013). Gaussian 09, Revision D.01. Wallingford, CT: Gaussian, Inc.

[B10] Han J. (2013). 1,3,4-Oxadiazole Based Liquid Crystals. J. Mater. Chem. C. 1, 7779–7797. 10.1039/c3tc31458h

[B11] Han J. Chui S. S. Y. Che C. M. (2006). Thermotropic Liquid Crystals Based on Extended 2,5-Disubstituted-1,3,4-Oxadiazoles: Structure-Property Relationships, Variable-Temperature Powder X-ray Diffraction, and Small-Angel X-ray Scattering Studies. Chem. Asian J. 1, 814–825. 10.1002/asia.200600252 17441124

[B12] Han J. Wang F.-L. Liu Y.-X. Zhang F.-Y. Meng J.-B. He Z.-J. (2012). Calix[4]arene-based 1,3,4-oxadiazole: Novel Fluorescent Chemosensors for Specific Recognition of Cu^2+^ . ChemPlusChem 77, 196–200. 10.1002/cplu.201200004

[B13] Han J. Wang Z.-Z. Wu J. Zhu L.-R. (2015). A Room-Temperature Liquid Crystalline Polymer Based on Discotic 1,3,4-oxadiazole. RSC Adv. 5, 47579–47583. 10.1039/c5ra05983f

[B14] Han J. Xi Z. Wang F. Bu L. Wang Y. (2018). Synthesis, Liquid Crystalline and Photoluminscent Properties of 1,3,4-oxadiazole Derivatives: from Calamitic Monomers, H-Shaped Dimers to Calix[4]arene-Based Tetramers. Dyes Pigm. 154, 234–241. 10.1016/j.dyepig.2018.03.008

[B15] Hobzova R. Sysel P. Duskova-Smrckova M. (2010). Synthesis and Characterization of Calix[4]arene-Containing Polyimides. Polym. Int. 60, 405–413. 10.1002/pi.2962

[B16] Kamel A. H. Amr A. E.-G. E. Almehizia A. A. Elsayed E. A. Moustafa G. O. (2021). Low-cost Potentiometric Paper-Based Analytical Device Based on Newly Synthesized Macrocyclic Pyrido-Pentapeptide Derivatives as Novel Ionophores for point-of-care Copper(II) Determination. RSC Adv. 11, 27174–27182. 10.1039/d1ra04712d PMC9037668 35480650

[B17] Kim H. J. Min Hee Lee M. H. Mutihac L. Vicens J. Kim J. S. (2012). Host–guest Sensing by Calixarenes on the Surfaces. Chem. Soc. Rev. 41, 1173–1190. 10.1039/c1cs15169j 21870018

[B18] Kowser Z. Rayhan U. Akther T. Redshaw C. Yamato T. (2021). A Brief Review on Novel Pyrene Based Fluorometric and Colorimetric Chemosensors for the Detection of Cu^2+^ . Mater. Chem. Front. 5, 2173–2200. 10.1039/D0QM01008A

[B19] Kumar M. Kumar N. Bhalla V. (2012). Ratiometric Nanomolar Detection of Cu^2+^ Ions in Mixed Aqueous media: a Cu^2+^/Li^+^ Ions Switchable Allosteric System Based on Thiacalix[4]crown. Dalton Trans. 41, 10189–10193. 10.1039/c2dt31081c 22847009

[B20] Liu S. Wang Y.-M. Han J. (2017). Fluorescent Chemosensors for Copper(II) Ion: Structure, Mechanism and Application. J. Photochem. Photobio. C. 32, 78–103. 10.1016/j.jphotochemrev.2017.06.002

[B21] Liu S. Wu Q. Zhang T. Zhang H. Han J. (2021). Supramolecular brush Polymers Prepared from 1,3,4-oxadiazole and Cyanobutoxy Functionalized Pillar[5]arene for Detecting Cu^2+^ . Org. Biomol. Chem. 19, 1287–1291. 10.1039/d0ob02587a 33508056

[B22] Lvova L. Caroleo F. Garau A. Lippolis V. Giorgi L. Fusi V. (2018). A Fluorescent Sensor Array Based on Heteroatomic Macrocyclic Fluorophores for the Detection of Polluting Species in Natural Water Samples. Front. Chem. 6, 258. 10.3389/fchem.2018.00258 30003078 PMC6032370

[B23] Ma J. Song M. Boussouar I. Tian D. Li H. (2015). Recent Progress of Calixarene-Based Fluorescent Chemosensors towards Mercury Ions. Supramole. Chem. 27, 444–452. 10.1080/10610278.2014.988627

[B24] Meng X. Wang P. Bai R. He L. (2020). Blue-green-emitting Cationic Iridium Complexes with Oxadiazole-type Counter-anions and Their Use for Highly Efficient Solution-Processed Organic Light-Emitting Diodes. J. Mater. Chem. C 8, 6236–6244. 10.1039/d0tc01054e 32558861

[B25] Miranda A. S. Serbetci D. Marcos P. M. Ascenso J. R. Berberan-Santos M. N. Hickey N. (2019). Ditopic Receptors Based on Dihomooxacalix[4]arenes Bearing Phenylurea Moieties with Electron-Withdrawing Groups for Anions and Organic Ion Pairs. Front. Chem. 7, 758. 10.3389/fchem.2019.00758 31781541 PMC6857623

[B26] Ni X.-L. Cong H. Yoshizawa A. Rahman S. Tomiyasu H. Rayhan U. (2013). Heteroditopic Thiacalix[4]arene Receptor Having Ester and Bipyridyl Moieties for Ions Binding with Positive/negative Allosteric Effect. J. Mol. Struc. 1046, 110–115. 10.1016/j.molstruc.2013.04.040

[B27] Noruzi E. B. Kheirkhahi M. Shaabani B. Geremia S. Hickey N. Asaro F. (2019). Design of a Thiosemicarbazide-Functionalized Calix[4]arene Ligand and Related Transition Metal Complexes: Synthesis, Characterization, and Biological Studies. Front. Chem. 7, 663. 10.3389/fchem.2019.00663 31649917 PMC6794423

[B28] Ömeroğlu İ. Tümay S. O. Makhseed S. Husain A. Durmuş M. (2021). A Highly Sensitive “ON–OFF–ON” Dual Optical Sensor for the Detection of Cu(II) Ion and Triazole Pesticides Based on Novel BODIPY-Substituted Cavitand. Dalton Trans. 50, 6437–6443. 10.1039/d1dt00792k 33890599

[B29] Sheldrick G. M. (1997). SHELXS-97 and SHELXL-97. Gottingen: University of Gottingen.

[B30] Singh G. Shilpy Singh A. Diksha Pawan (2020). Synthesis of Organosilocane Allied N-Heteroaryl Schiff Base Chemosensor for the Detection of Cu^2+^ Metal Ions and Their Biological Applications. New J. Chem. 44, 13542–13552. 10.1039/d0nj01774d

[B31] Sivaraman G. Iniy M. Anand T. Kotla N. G. Sunnapu O. Singaravadivel S. (2018). Chemically Diverse Small Molecule Fluorescent Chemosensors for Copper Ion. Coord. Chem. Rev. 357, 50–104. 10.1016/j.ccr.2017.11.020

[B32] Thordarson P. (2011). Determining Association Constants from Titration Experiments in Supramolecular Chemistry. Chem. Soc. Rev. 40, 1305–1323. 10.1039/c0cs00062k 21125111

[B33] Turski M. L. Thiele D. J. (2009). New Roles for Copper Metabolism in Cell Proliferation, Signaling, and Disease. J. Biol. Chem. 284, 717–721. 10.1074/jbc.R800055200 18757361 PMC2613604

[B34] Udhayakumari D. Naha S. Velmathi S. (2017). Colorimetric and Fluorescent Chemosensors for Cu^2+^. A Comprehensive Review from the Years 2013-15. Anal. Methods 9, 552–578. 10.1039/c6ay02416e

[B35] Wang L. Gong X. Bing Q. Wang G. (2018). A New Oxadiazole-Based Dual-Mode Chemosensor: Colorimetric Detection of Co^2+^ and Fluorometric Detection of Cu^2+^ with High Selectivity and Sensitivity. Microchem. J. 142, 279–287. 10.1016/j.microc.2018.07.008

[B36] Xie D.-H. Wang X.-J. Sun C. Han J. (2016). Calix[4]arene Based 1,3,4-oxadiazole as a Fluorescent Chemosensor for Copper(II) Ion Detection. Tetrahedron Lett. 57, 5834–5836. 10.1016/j.tetlet.2016.11.051

[B37] Zhang Z. Liu Y. Wang E. (2019). A Highly Selective “Turn-on” Fluorescent Probe for Detecting Cu^2+^ in Two Different Sensing Mechanisms. Dyes Pigm. 163, 533–537. 10.1016/j.dyepig.2018.12.039

