# Supplementary material for: A Novel Calix[4]Crown-Based 1,3,4-Oxadiazole as a Fluorescent Chemosensor for Copper(II) Ion Detection
Source: Front Chem. 2021 Nov 12;9:766442. doi: 10.3389/fchem.2021.766442 (PMC8632693; doi:10.3389/fchem.2021.766442)
Supplement: Supplementary file 1 [file Table1.DOCX]

Supplementary Material

**Calix[4]crown-based 1,3,4-Oxadiazoles as Fluorescent Chemosensors for copper(II) ion detection**

**Chun Sun^1^, Siyi Du^1^, Tianze Zhang^1^, Jie Han^1^***

^1^ Key Laboratory of Advanced Energy Materials Chemistry (Ministry of Energy), College of Chemistry, Nankai University, Tianjin 300071, China

*** Correspondence:**
Jie Han
[hanjie@nankai.edu.cn](mailto:hanjie@nankai.edu.cn)

| Page | Table of Contents |
| --- | --- |
| S2 | Experimental detail for the synthesis of compound **2** and **3** |
| S3 | **Figure S1** ^1^H NMR, ^13^C NMR spectra of **4** |
| S4 | **Figure S2** ^1^H NMR, ^13^C NMR spectra of **5** |
| S5 | **Figure S3** ^1^H NMR, ^13^C NMR spectra of **6** |
| S6 | **Figure S4** ^1^H NMR, ^13^C NMR spectra of **1** |
| S7 | **Figure S5** HRMS spectrum of **1** |
| S7 | **Figure S6** Fluorescence titration of **1** (1.0 × 10^−5^ mol/L) with Cu^2+^ ion (0-2 equiv) (λ_exc_ = 334 nm, Slit = 2.5) |
| S8 | **Table S1** X-ray crystallographic data of compound **5** |
| S9 | Cartesian Coordinates for Optimized Structures of **1** and **1·Mg^2+^** |
| S19 | Reference |

# Synthesis of Compounds

1.1 Synthesis of 25.27-dihydroxy-26,28-di-n-propoxycalix[4]arene (**2**).^[1]^

Under N_2_ atmosphere, to a suspension of calix[4]arene (9.71 g, 22.8 mmol) in anhydrous CH_3_CN (60 mL) were added 1-iodopropane (8.56 g, 50.4 mmol) and K_2_CO_3_ (12.64 g, 91.6 mmol), and the reaction mixture was refluxed with stirring for 24 h. After the mixture cooled to the room temperature, the solvent was evaporated under reduced pressure, the residue was taken up with CH_2_C1_2_ (100 mL), and the organic phase was washed sequentially with aqueous HC1(30 mL × 3), water (30 mL × 3) and brine. The organic solution was dried with anhydrous MgSO_4_ and filtered. Evaporation of the organic solvent under reduced pressure afforded the crude solid which was recrystallized (CH_2_Cl_2_-EtOH) and gave 58% yield of **2** as white crystals. ^1^H NMR (400 MHz, CDCl_3_) δ 8.32 (s, 2H), 7.05 (d, *J* = 8.0 Hz, 4H), 6.93 (d, *J* = 8.0 Hz, 4H), 6.75 (t, *J* = 8.0 Hz, 2H), 6.64 (t, *J* = 8.0 Hz, 2H), 4.32 (d, *J* = 12.9 Hz, 4H), 3.98 (t, *J* = 6.2 Hz, 4H), 3.38 (d, *J* = 12.9 Hz, 4H), 2.10 – 2.05 (m, 4H), 1.32 (t, *J* = 7.4 Hz, 6H).

1.2 Synthesis of **3**^[2]^

To a solution of compound **2** (9.18 g, 18 mmol) in CHC1_3_ (45 mL) was added dropwise a solution of Br_2_ (2 mL, 40 mmol) in CHC1_3_ (5 mL) during 1 h at 0 °C. After being stirred for 3 h at room temperature the precipitate formed was filtered off and washed with cold CHC1_3_ to afford **3** as white solid in 82% yield. ^1^H NMR (400 MHz, CDCl_3_) δ 8.40 (s, 2H), 7.16 (s, 4H), 6.94 (d, *J* = 8.0 Hz, 4H), 6.81 (t, *J* = 8.0 Hz, 2H), 4.25 (d, *J* = 13.0 Hz, 4H), 3.95 (t, *J* = 5.9 Hz, 4H), 3.32 (d, *J* = 13.0 Hz, 4H), 2.10 – 2.01 (m, 4H), 1.29 (t, *J* = 7.0 Hz, 6H).

# Structural Characterization

2.1 **Figure S1** ^1^H NMR, ^13^C NMR spectra of **4**

^1^H NMR (400 MHz, CDCl_3_) of **4**

^13^C NMR (101 MHz, CDCl_3_) of **4**

2.2 **Figure S2** ^1^H NMR, ^13^C NMR spectra of **5**

^1^H NMR (400 MHz, CDCl_3_) of **5**

^13^C NMR (101 MHz, CDCl_3_) of **5**

2.3 **Figure S3** ^1^H NMR, ^13^C NMR spectra of **6**

^1^H NMR (400 MHz, CDCl_3_) of **6**

^13^C NMR (101 MHz, DMSO-*d_6_*) of **6**


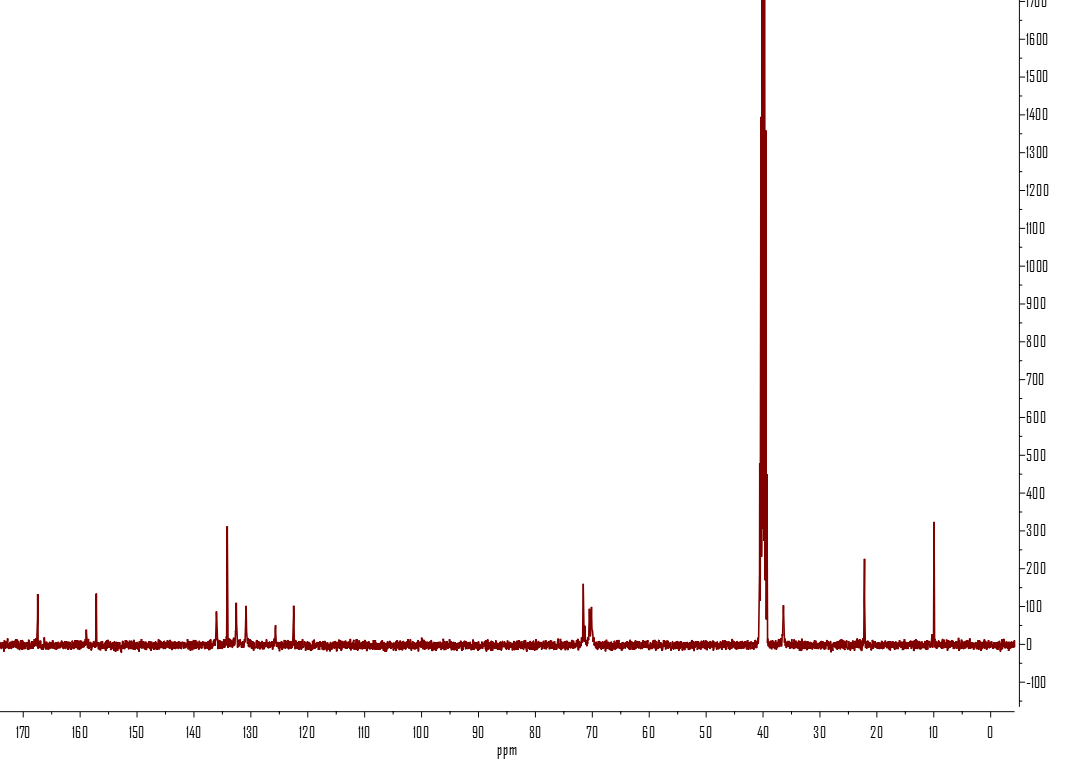


2.4 **Figure S4** ^1^H NMR, ^13^C NMR spectra of **1**

^1^H NMR (400 MHz, CDCl_3_) of **1**

^13^C NMR (101 MHz, CDCl_3_) of **1**

2.5 **Figure S5** HRMS spectrum of **1**


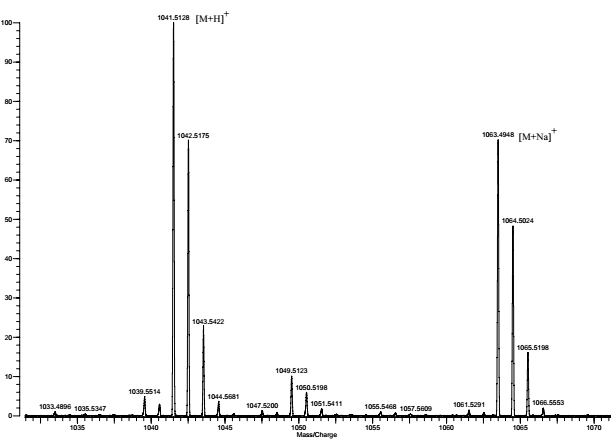


2.6 **Figure S6** Fluorescence titration of **1** (1.0 × 10^−5^ mol/L) with Cu^2+^ ion (0-2 equiv) (λ_exc_ = 334 nm, Slit = 2.5)





# Crystallographic Data and Structural Information

**Table S1** X-ray crystallographic data of compound **5**

| Compound | **5** |
| --- | --- |
| Empirical formula | C_45_H_49_Cl_3_N_2_O_7_ |
| Formula weight | 836.21 |
| Temperature / K | 293 |
| Crystal system | Orthorhombic |
| Space group | *P*nma |
| *a* [Å] | 28.244(6) |
| *b* [Å] | 15.396(3) |
| *c* [Å] | 10.072(2) |
| *α* [deg] | 90 |
| *β* [deg] | 90 |
| *γ* [deg] | 90 |
| Volume [Å^3^] | 4379.8(15) |
| *Z* | 4 |
| *ρ_calc_* [g cm^-3^] | 1.268 |
| *μ* [mm^-1^] | 0.260 |
| *F*(000) | 1760.0 |
| Reflections collected | 19950 |
| Unique / parameters | 2392 / 282 |
| *R*(int) | 0.2139 |
| Completeness to theta = 27.48 | 99.8 % |
| Max. / min. transmission | 0.969 / 0.957 |
| Goodness-of-fit on *F^2^* | 1.141 |
| *R*_1_*,* *wR*_2_ [*I*>2*σ*(*I*)] | 0.1282, 0.2412 |
| *R*_1_*,* *wR*_2_ (all data) | 0.1977, 0.2776 |
| CCDC no. | 2106744 |

# Cartesian Coordinates for Optimized Structures of 1 and 1·Mg^2+^

The chemosensor **1**

C 0.19701700 3.60687500 -0.91209500

C -0.05121600 3.32060400 0.43448100

H 0.72173900 3.51237400 1.17268000

C -1.27643400 2.79536300 0.84770700

C -2.27139300 2.56668500 -0.12060700

C -2.03304000 2.81056800 -1.48967700

C -0.79956300 3.34238900 -1.86015700

H -0.59323500 3.55526200 -2.90440900

C -3.07568600 2.57510200 -2.57576800

H -2.57444600 2.72978000 -3.53899200

H -3.83453600 3.36648300 -2.51995800

C -3.80957500 1.24370600 -2.59281900

C -5.20966900 1.23506700 -2.60581300

H -5.74344400 2.18286500 -2.59969100

C 0.11645000 -3.65009800 -0.89566100

C -0.13131900 -3.35577300 0.44968900

H 0.63494900 -3.55949800 1.19160700

C -1.34934600 -2.80993000 0.85567000

C -2.33666800 -2.56478500 -0.11731600

C -2.09711100 -2.81281700 -1.48472200

C -0.87196400 -3.37014300 -1.84714200

H -0.66582200 -3.59048400 -2.88984200

C -3.11991700 -2.54526100 -2.58343800

H -2.59746000 -2.67499700 -3.53923500

H -3.88447800 -3.33316000 -2.56274300

C -3.84069700 -1.20714600 -2.58651400

C -5.23921800 -1.17079700 -2.59751700

H -5.79434700 -2.10609900 -2.58820100

C -3.13601900 0.00941500 -2.63494400

C -1.18648200 0.09341200 -4.00287000

H -1.77664700 -0.49907800 -4.71624400

H -1.23342800 1.14416300 -4.32484600

C 1.16222400 0.39336500 -3.01873700

H 2.17653700 -0.02135300 -3.02528300

H 1.23256000 1.44993700 -3.30237200

H 0.77644500 0.34128600 -1.99633400

C 0.25892100 -0.38881400 -3.97577600

H 0.27163400 -1.45207500 -3.71013500

H 0.64426200 -0.31645900 -5.00236800

C -1.54541500 -0.00312800 2.39628000

C 0.82272600 -0.05255500 2.25128600

H 0.88248400 -0.95004800 2.88630100

H 0.91493100 0.81979900 2.91691100

C 1.93432500 -0.05585300 1.21154400

H 1.79044300 -0.91789900 0.54991300

H 1.83135600 0.83902800 0.58682700

C 3.32590300 -0.10153400 1.85073400

H 3.49932500 0.76529100 2.50072600

C -1.51040600 2.56154200 2.33410200

H -2.17339400 3.35049400 2.71406100

H -0.54936300 2.72626500 2.83902000

C -2.09287400 1.23152000 2.78658400

C -3.18531400 1.22606100 3.66095700

H -3.60356800 2.17540000 3.98865900

H 3.45973800 -1.00468900 2.45927600

H 4.10704600 -0.10113800 1.08359900

C -1.59021600 -2.57012900 2.33971600

H -2.28627800 -3.33435900 2.71072400

H -0.64176500 -2.77106300 2.85518900

C -2.12945700 -1.22036000 2.78849500

C -3.22168300 -1.17950000 3.66254300

H -3.66875700 -2.11513600 3.99159000

C -3.74043200 0.03162600 4.11383800

H -4.58378600 0.04468800 4.79903200

C -4.56690300 2.92176100 0.46131500

H -4.84666300 3.39432000 -0.48883300

H -4.31304600 3.71988800 1.17254500

C -5.73209500 2.08587800 0.97377700

H -5.85974200 1.21175700 0.32651200

H -5.52559900 1.72410900 1.99011000

C -7.97280900 2.47402000 1.73090900

H -8.66846200 3.32018100 1.76620500

H -7.65415300 2.24707500 2.76147800

C -8.72248700 1.26411300 1.17850600

H -9.73663400 1.24704900 1.61636300

H -8.83269800 1.36520300 0.08641900

C -4.64615900 -2.87094900 0.43791600

H -4.93362500 -3.31319900 -0.52427800

H -4.41743500 -3.69169700 1.13185500

C -5.79032600 -2.01412000 0.96302500

H -5.88234300 -1.11806200 0.34019000

H -5.58420200 -1.68806200 1.99161700

C -8.05203200 -2.34678200 1.68166000

H -8.77422600 -3.17114000 1.68972300

H -7.73927500 -2.15287700 2.72073600

C -8.75713800 -1.10212300 1.14817100

H -9.77402300 -1.06193000 1.57806800

H -8.86078600 -1.17683400 0.05334900

O -1.75663200 -0.03742500 -2.68742600

O -0.43710100 -0.01717300 1.56955500

O -3.46878600 2.02421100 0.29068500

O -6.88264500 2.92259300 0.95166600

O -3.52364800 -1.99968000 0.29374000

O -6.96673800 -2.81203500 0.90543100

O -8.04043600 0.06627500 1.51379100

C -5.92586200 0.04079400 -2.62558600

H -7.01226000 0.05444200 -2.64874600

C 1.38150600 -4.22385400 -1.33051900

O 2.35330000 -4.49919000 -0.40525600

C 3.36740200 -5.01168900 -1.17032600

C 1.47215600 4.15383900 -1.35127000

O 2.43246500 4.45951100 -0.42407500

C 3.46164300 4.93345200 -1.19398900

N 3.04852900 -5.04584600 -2.43439400

N 1.76144100 -4.53426400 -2.53659400

N 3.16139900 4.91892000 -2.46295300

N 1.87223700 4.41201300 -2.56337100

C 4.60148600 -5.43306700 -0.53395000

C 5.64653000 -5.95635200 -1.31418000

C 4.79273300 -5.34252800 0.85294500

C 6.83501900 -6.36923900 -0.73541500

H 5.50958800 -6.03752200 -2.38779800

C 5.97938100 -5.75414700 1.44272800

H 3.99887400 -4.94778300 1.47938700

C 7.04155300 -6.27323000 0.66377600

H 7.60999500 -6.77113800 -1.37656400

H 6.07856000 -5.67175800 2.51819200

C 4.68896800 5.37168600 -0.55602000

C 5.75506000 5.84286400 -1.34092000

C 4.85277900 5.34894600 0.83719100

C 6.93737200 6.27080000 -0.76042800

H 5.63976200 5.87109100 -2.41977000

C 6.03289500 5.77636300 1.42871200

H 4.04232200 4.99572700 1.46716300

C 7.11591600 6.24369400 0.64565500

H 7.72943900 6.63049800 -1.40575400

H 6.11048700 5.74722900 2.50871500

N 8.23434200 -6.66619000 1.24291900

N 8.30160500 6.65283700 1.22777500

C 9.33768800 7.25972800 0.40885300

H 9.00215000 8.18903600 -0.07660800

H 10.19787200 7.49546300 1.03785000

H 9.67815700 6.57161900 -0.37489500

C 8.39930900 6.75130600 2.67413600

H 7.68511500 7.47471000 3.09706700

H 8.21978800 5.77967600 3.15149300

H 9.40741800 7.07080300 2.94373900

C 8.35933800 -6.69418100 2.69025100

H 7.64862500 -7.39021900 3.16227100

H 9.37039600 -7.00766900 2.95589100

H 8.19585900 -5.69876300 3.12203200

C 9.24604800 -7.32834600 0.43642500

H 8.89036900 -8.27821200 0.00837800

H 9.57720400 -6.68699000 -0.38974600

H 10.11685700 -7.54045900 1.05922300

The complex **1·Mg^2+^**

C 3.93148100 0.27260700 1.00191200

C 3.64867100 -0.06952200 -0.33212600

H 4.05360700 0.54186300 -1.13267400

C 2.87606100 -1.18950500 -0.63918600

C 2.39607400 -1.95942700 0.43634800

C 2.64079000 -1.65051300 1.78764000

C 3.41740300 -0.51942100 2.04193500

H 3.65007900 -0.23640300 3.06384700

C 2.19754100 -2.49098700 2.99011200

H 2.13606000 -1.80370500 3.84282100

H 3.01964600 -3.17351700 3.23923200

C 0.92052600 -3.32257800 2.95224900

C 0.95781300 -4.68170000 3.28194500

H 1.90731300 -5.14217400 3.54062000

C -4.15715300 0.28291300 0.68646600

C -3.66335100 0.14121300 -0.62000400

H -3.88171000 0.91162600 -1.35306600

C -2.90892200 -0.97419200 -0.99780800

C -2.66486800 -1.94177400 -0.00909100

C -3.13651900 -1.84074900 1.31643700

C -3.88631200 -0.71554100 1.64230100

H -4.29005100 -0.59169400 2.64246800

C -2.92814800 -2.93938800 2.35546700

H -3.34187300 -2.57262900 3.30301900

H -3.56809700 -3.78919700 2.08783300

C -1.53648900 -3.51345200 2.63418200

C -1.43343500 -4.87430000 2.96187100

H -2.33239600 -5.48462000 2.96957600

C -0.34113800 -2.77040500 2.65910600

C -0.85069100 -0.54499100 3.42526400

H -1.88585600 -0.83647400 3.60339400

H -0.24531500 -0.83935700 4.28775100

C 0.65247800 1.54063600 3.29182200

H 0.63281700 2.61691700 3.09515400

H 1.03117400 1.40516200 4.31112700

H 1.37468700 1.08729300 2.60682600

C -0.74776800 0.94031900 3.14538200

H -1.18417800 1.15180400 2.16341600

H -1.42165300 1.41694100 3.86971900

C 0.07632100 -1.42191800 -2.26628400

C 0.28999100 0.86065900 -1.49357000

H -0.33743000 1.07209200 -2.36412500

H 1.34001300 0.93434800 -1.78808100

C -0.02817500 1.78008200 -0.33666300

H -1.07571400 1.64276200 -0.04526700

H 0.59906500 1.51400900 0.52014200

C 0.22221400 3.24585400 -0.72303400

H 1.26970500 3.41639100 -0.99426600

C 2.67668200 -1.57870400 -2.10072000

H 3.34895900 -2.41832100 -2.32016600

H 3.06599400 -0.75133400 -2.70761000

C 1.30805900 -1.95977000 -2.66956600

C 1.27314600 -2.86436800 -3.74238100

H 2.20405600 -3.29828000 -4.09752400

H -0.40239600 3.55188900 -1.56943900

H -0.01327600 3.90418500 0.11773600

C -2.47973200 -1.09651900 -2.46017300

H -3.27382600 -1.63242200 -2.99569200

H -2.50168700 -0.08272700 -2.88003100

C -1.15663600 -1.74447300 -2.86028500

C -1.12886200 -2.64770200 -3.92843500

H -2.05710300 -2.91155900 -4.42796100

C 0.07512500 -3.19963000 -4.36792300

H 0.07841300 -3.89328600 -5.20306700

C 2.41659000 -4.32590500 0.07419700

H 2.70569100 -4.57520900 1.09680400

H 3.31415800 -4.09535000 -0.50348000

C 1.65203800 -5.48060500 -0.54585800

H 0.69643600 -5.62384200 -0.03107800

H 1.44512200 -5.28698400 -1.60671900

C 2.38447200 -7.63720700 -1.33828200

H 3.23563700 -8.29661900 -1.14709400

H 2.48418200 -7.22366900 -2.35300700

C 1.09852900 -8.44398900 -1.25007600

H 1.20427500 -9.34672500 -1.87295600

H 0.92361900 -8.76835000 -0.21246000

C -2.65614200 -4.16786300 -0.90258500

H -3.45766900 -4.39408500 -0.19555900

H -3.09669500 -3.81031100 -1.83544600

C -1.80793100 -5.40256300 -1.14484900

H -1.27695500 -5.68255000 -0.22695500

H -1.06576200 -5.21654600 -1.93106700

C -2.25767900 -7.40575100 -2.41096400

H -3.14564600 -7.98246400 -2.68374000

H -1.84666800 -6.94456400 -3.32162400

C -1.21662400 -8.33909800 -1.81301200

H -1.11054200 -9.21943600 -2.46708100

H -1.54662000 -8.69126500 -0.82331200

O -0.36966500 -1.39843400 2.29651700

O 0.04006300 -0.55615600 -1.14379000

O 1.60530100 -3.09542800 0.11642000

O 2.49885000 -6.59683900 -0.36856400

O -1.86194800 -3.06747700 -0.32993000

O -2.73563600 -6.39871400 -1.52022800

O 0.02095900 -7.64517900 -1.72052500

C -0.20888700 -5.44749000 3.29607300

H -0.16072000 -6.49831600 3.56431700

C -4.92478000 1.44120300 1.09162200

O -5.31227800 2.37997900 0.17860400

C -5.99199100 3.29228600 0.95199500

C 4.72771600 1.43366800 1.34107900

O 5.35752700 2.15945100 0.37002400

C 5.99150900 3.14411400 1.09216700

N -5.99644000 2.92800000 2.21947800

N -5.31220400 1.74389600 2.29917000

N 5.74605500 3.01852000 2.38112500

N 4.93799400 1.92177600 2.53107000

C -6.58742300 4.45264600 0.35130400

C -7.27838300 5.37763400 1.16321900

C -6.51817100 4.70328400 -1.03297000

C -7.87097100 6.49859100 0.62016200

H -7.34313900 5.19669700 2.23123300

C -7.10887800 5.82238600 -1.58736700

H -5.99781800 4.00528100 -1.68192700

C -7.80810100 6.76100400 -0.77765600

H -8.39255300 7.18060300 1.27945200

H -7.03660600 5.97363200 -2.65694100

C 6.80393800 4.12500500 0.42776200

C 7.44321900 5.12599500 1.18991700

C 7.00448800 4.11953500 -0.96606600

C 8.24442500 6.07551000 0.59038100

H 7.30122200 5.14083300 2.26564600

C 7.80526600 5.06575900 -1.57678200

H 6.53002600 3.35685200 -1.57631300

C 8.45577600 6.07808900 -0.81724600

H 8.71855300 6.82280200 1.21375800

H 7.93665800 5.02195200 -2.65053900

N -8.39621500 7.86890500 -1.31982000

N 9.25253200 7.01442400 -1.41445400

C 9.92384300 8.03137200 -0.61096300

H 10.61487000 7.58257300 0.11405100

H 10.49898300 8.68306700 -1.26857500

H 9.20246100 8.65207400 -0.06507800

C 9.47222000 6.98351600 -2.85557000

H 9.94556800 6.04503100 -3.17278300

H 8.53146000 7.10169700 -3.40864200

H 10.13180000 7.80526500 -3.13431200

C -8.32893000 8.11041600 -2.75629400

H -8.81188600 7.30521400 -3.32530500

H -8.84449700 9.04262700 -2.98675400

H -7.29065300 8.20399400 -3.09973000

C -9.12415300 8.80708500 -0.47086100

H -9.96600600 8.32080200 0.03797000

H -8.46954500 9.25303500 0.28847300

H -9.52216200 9.61210600 -1.08845800

Mg -0.16566800 -2.01550000 0.29760700

# Reference

[1] Arduini, A., Fabbi, M., Mantovani, M., Mirone, L., Pochini, A., Secchi, A. and Ungaro, R. Calix[4]arenes blocked in a rigid cone conformation by selective functionalization at the lower rim, *J.Org.Chem.*1995, 60, 1454-1457.

[2] Hobzova, R., Sysel, P., and Duskova-Smrckova, M. Synthesis and characterization of calix[4]arene-containing polyimides. *Polym. Int*. 2010, 60, 405-413.
